# Kinematic Analysis and Application to Control Logic Development for RHex Robot Locomotion

**DOI:** 10.3390/s24051636

**Published:** 2024-03-02

**Authors:** Piotr Burzyński, Ewa Pawłuszewicz, Leszek Ambroziak, Suryansh Sharma

**Affiliations:** 1 Department of Industrial Process Automation, Faculty of Mechanical Engineering, Bialystok University of Technology, Wiejska 45C, 15-351 Bialystok, Poland; p.burzynski@doktoranci.pb.edu.pl (P.B.); e.pawluszewicz@pb.edu.pl (E.P.); l.ambroziak@pb.edu.pl (L.A.); 2 Networked Systems Group, Delft University of Technology, Building 28, Van Mourik Broekmanweg 6, 2628 XE Delft, The Netherlands

**Keywords:** C-legged hexapod, mobile robot, walking robot, kinematics modeling, simulation

## Abstract

This study explores the kinematic model of the popular RHex hexapod robots which have garnered considerable interest for their locomotion capabilities. We study the influence of tripod trajectory parameters on the RHex robot’s movement, aiming to craft a precise kinematic model that enhances walking mechanisms. This model serves as a cornerstone for refining robot control strategies, enabling tailored performance enhancements or specific motion patterns. Validation conducted on a bespoke test bed confirms the model’s efficacy in predicting spatial movements, albeit with minor deviations due to motor load variations and control system dynamics. In particular, the derived kinematic framework offers valuable insights for advancing control logic, particularly navigating in flat terrains, thereby broadening the RHex robot’s application spectrum.

## 1. Introduction

The domain of mobile robotics has witnessed a notable evolution in recent years, driven by technological advancements and an ever-evolving array of applications across various contexts. This evolution has been paralleled by substantial research endeavors aimed at mitigating electronic costs [1], harnessing the augmented computational capabilities of microchips, and revolutionizing intelligent and adaptable manufacturing processes [2]. Considerable endeavors extend to the refinement of sophisticated control strategies [3] and the enhancement of autonomous navigation capabilities, alongside the development of measurement techniques and sensors resilient to noise interference [4,5]. Furthermore, the integration of a broad spectrum of artificial intelligence methodologies, encompassing machine learning and neuromorphic control systems, is being pursued at multiple tiers within the robot’s architecture [6,7]. Mobile robots, classified according to their operational domains: ground (Unmanned Ground Vehicles, UGVs), aerial (Unmanned Aerial Vehicles, UAVs), aquatic—submersible (Autonomous Underwater Vehicles, AUVs), and surface-based (Unmanned Surface Vehicles, USVs) [8], have extended their applicability beyond traditional settings, adeptly adapting to intricate terrains and even modifying their morphology and locomotion to optimize efficiency [9]. The 1980s marked a turning point with the introduction of dynamic locomotion in robots, significantly advanced by research at Tokyo University and MIT’s LegLab [10,11]. The field further evolved with Honda’s P2 humanoid in the 1990s, demonstrating greater versatility and leading to broader commercial and research interest. Recent decades have focused on enhancing the dynamic walking and running capabilities of legged robots, with ongoing challenges in improving efficiency, speed, and robustness [12,13].

Within the realm of land-based robotics, the distinction among wheeled, tracked, and legged configurations underscores a trade-off between velocity and terrain adaptability. Wheeled robots exhibit superior speed and energy efficiency on well-defined terrains, yet encounter challenges in rugged, obstacle-laden environments where legged robots, with their capacity to navigate discrete footholds and utilize additional appendages for stability, excel [14]. Despite the inherent complexities associated with their intricate mechanics [15], propulsion [16], and control systems [17], legged robots offer unparalleled versatility in natural and unstructured environments, drawing inspiration from various animal locomotion mechanisms to achieve dynamic stability and mobility [18,19]. Single-legged robots are inspired by saltatorial animals (animals that locomote by jumping) [20], two-legged robots by humanoids [21], four legs on quadrupeds [22], and more than four legs are inspired by insects [23,24,25].

This paper delves into the hexapod configuration, particularly focusing on the RHex robot, which embodies a fusion of stability and adaptability. Hexapod robots, celebrated for their static stability and versatility, exemplify the ongoing research pursuit to harmonize mobility, efficiency, and intricacy [26]. Through an examination of the RHex robot, this study contributes to a nuanced comprehension of legged robotics, proposing innovations in leg design and control methodologies to enhance its functionality across diverse terrains, including aquatic environments, as demonstrated by the flapped-paddle amphibian variant-FLHex [27]. Our exploration of the kinematics and control mechanisms of the RHex robot endeavors to push the boundaries of attainable feats in legged robotics, thereby establishing a new standard for adaptability and performance in mobile robotic applications.

The RHex hexapod robot introduces an innovative departure from the traditional multi-segmented, multi-degree-of-freedom leg configuration commonly found in legged robots, opting instead for a singular C-shaped leg with a solitary degree of freedom per leg [23,28]. This design ingeniously strikes a balance between the intricacies of legged locomotion and the efficiency of wheeled mobility, endowing the RHex with the capability to traverse uneven terrains with remarkable stability and resilience. The robot’s legs, capable of high-speed synchronized rotations, facilitate a broad spectrum of mobility tasks, encompassing navigating slopes [29] and stairs [30,31], overcoming obstacles [27], and executing intricate maneuvers such as flipping [32].

**Figure 1:**
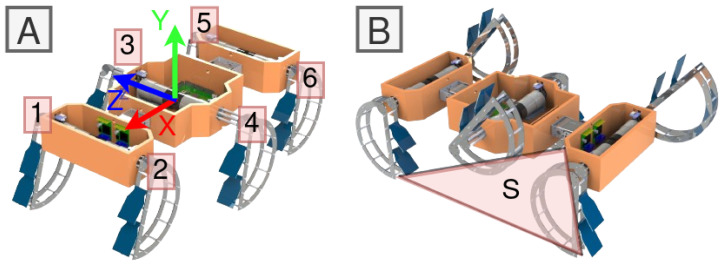
A flapped-paddle amphibian variant of the RHex robot, FLHex (Video [34]) showing (**A**) center of mass and coordinate axes of the robot and (**B**) alternating tripod pairs where tripod legs that are in contact with ground define a support triangle S.

**Figure 2:**
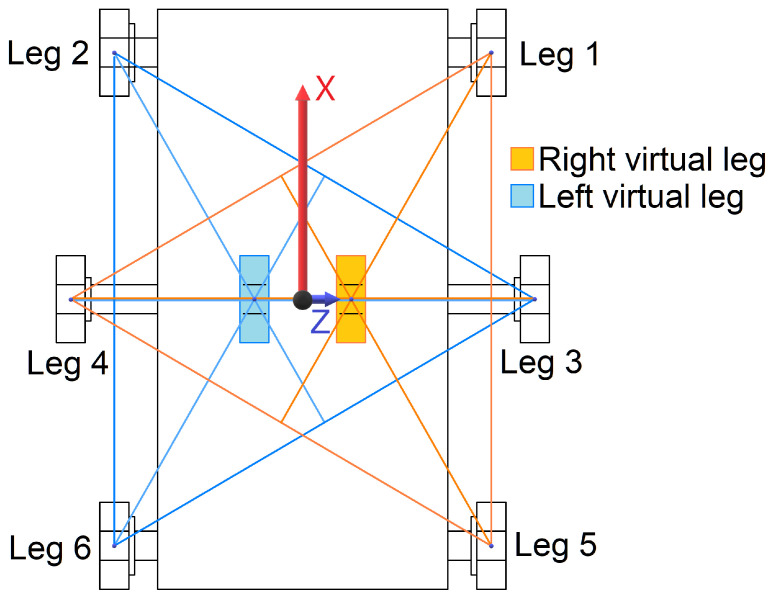
FLHex robot tripods acting as virtual legs.

Among the array of gaits employed by RHex robots, the alternating tripod gait emerges as particularly notable for its efficiency and dynamic stability, drawing inspiration from the locomotive patterns observed in insects such as cockroaches and beetles. This gait organizes the robot’s legs into two tripods—front and rear legs on one side paired with the middle leg on the opposite side—ensuring continuous ground contact for one tripod while the other repositions for the subsequent step [33]. This methodology not only facilitates efficient forward movement but also enhances the robot’s dynamic stability, as depicted by the support triangle illustrated in Figure 1B. The utilization of the alternating tripod gait ensures the continued placement of the robot’s center of gravity inside the support triangle, as demonstrated in Figure 2.

Further simplifying the complex design of the RHex, the concept of ’virtual legs’ combines the physical legs within each tripod, effectively transforming the robot’s structure into a body flanked by two composite legs. This abstraction plays a pivotal role in the development of control algorithms and mathematical models for the RHex, streamlining the approach to robot dynamics and control [35]. The adaptability and efficiency demonstrated by the RHex’s gait, validated through practical implementations and captured in video demonstrations [34], underscore the advanced design of the robot and its potential for diverse applications.

The principal objective of this investigation is to elucidate the dynamics governing the motion of the RHex robot, particularly when employing the alternating tripod gait—a gait of considerable significance and prevalence among RHex robots. We undertake an in-depth exploration into the development of a kinematic model specifically tailored to this gait, detailing the impact of various parameters on the robot’s spatial displacement for a predetermined number of steps. This model serves as a fundamental tool for refining the control and navigation of the RHex robot, offering insights into its operational capabilities.

The structure of this manuscript is organized to facilitate the comprehension of our findings and methodologies. Section 2 introduces an incremental model that focuses on a single C-shaped leg, mirroring the crawling gait of the RHex robot. Subsequently, Section 3 delves into the temporal facets of the leg trajectories integral to the alternating tripod gait. In Section 4, we extend the model to incorporate dual C-shaped legs, reflecting the walking gait of the RHex robot. Section 5 is devoted to the empirical validation of our model, drawing upon data derived from rigorous testing conducted on a specially constructed experimental setup. The ensuing analysis in Section 6 leverages the model to dissect how the intricacies of tripod trajectories and the nuances of the robot’s design influence a single gait cycle. This analysis culminates in the formulation of a comprehensive set of design principles aimed at optimizing the control of RHex robots. The manuscript concludes in Section 7, wherein we delineate our findings and discuss their implications for the field.

## 2. The Kinematics of a Single RHex Robot Leg

**Figure 3:**
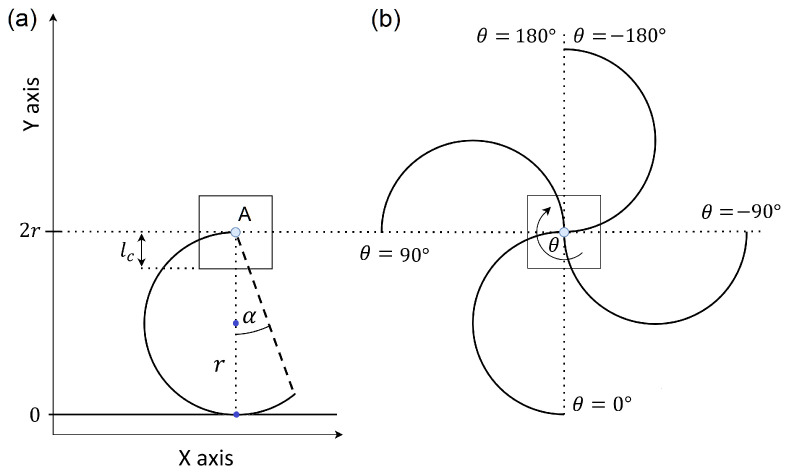
RHex robot C-leg kinematics (**a**) when positioned in the most upright position, (**b**) with varying value of θ as the leg rotates.

Let us start by considering a simplified system of one of the legs of RHex robots shown in Figure 3. The square represents the chassis of the robot, connected to the end of the robot’s leg (C-shaped curve) at its point of rotation located in the middle of the square (Point A in Figure 3). The leg shown as a circular arc with radius *r* and central angle 180°+α, where α describes the elongated part of the leg, with $\alpha \in \left[0{}^{\circ},90{}^{\circ}\right]$. In most RHex robots, the legs are in the shape of a semicircle $\left(\alpha =0{}^{\circ}\right)$ [1]. However, there are RHex robot designs with an extended leg $\left(\alpha >0{}^{\circ}\right)$ or even a fully circular leg that is used to increase the smoothness of the robot’s movement [35]. The chassis has only two degrees of freedom and it can displace only in the X-axis and Y-axis directions. The position of the robot’s leg is described by the angle $\theta \in \left[-180{}^{\circ},180{}^{\circ}\right]$ between the leg’s diameter at its current position and the leg’s diameter in the upright position of the leg (see Figure 3a). The system describing the absolute position of the leg is shown in Figure 3b.

**Figure 4:**
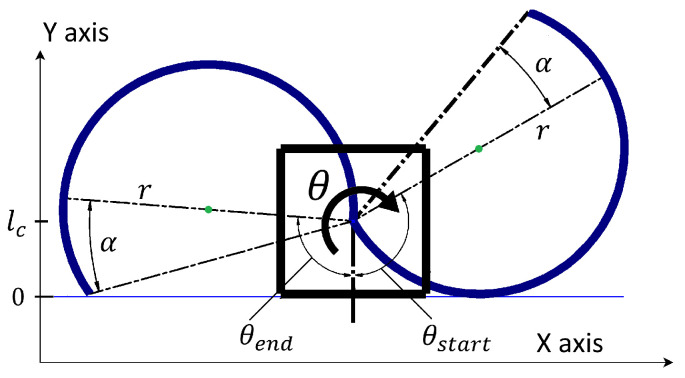
The robot leg positions at $\theta_{start}$, and $\theta_{end}$ showing when the leg-ground contact changes.

The maximum position on the Y-axis corresponds to the leg’s most upright position, equivalent to the leg’s diameter. The minimum position on the Y-axis is the distance from the robot’s leg rotational joint (pivot) to the bottom of the chassis represented by $l_{c}$. When the Y-axis position of the pivot is $l_{c}$, the robot’s chassis is in contact with the ground, the legs rotate in the air, and the system becomes stationary, as illustrated in Figure 4. The system, consisting of a body equipped with a single rotating C-shaped leg, initiates movement when the leg makes contact with the ground at position $\theta_{start}$ and returns to a stationary state when the leg breaks contact with the ground at position $\theta_{end}$. These positions, $\theta_{start}$ and $\theta_{end}$, are depicted in Figure 4. We see their dependence on the design parameters of the robot and its leg. The value of $\theta_{start}$ is determined by *r*, $l_{c}$ and the value of $\theta_{end}$ is influenced by *r*, $l_{c}$, and α. These can be calculated using the equations shown below.

For $\theta_{start}$

(1) $$1+\cos\left(\theta_{start\;}\right)=\frac{l_{c}}{r}$$

(2) $$\cos\left(\frac{\theta_{start}}{2}\right)=\pm \sqrt{\frac{l_{c}}{2r}}$$

where ± becomes − due to the leg being located in the II or III quadrant of the coordinate system (see Figure 3b), so

(3) $$\cos\left(\frac{\theta_{start\;}}{2}\right)=-\sqrt{\frac{l_{c}}{2r}}$$

(4) $$\theta_{start\;}=90{}^{\circ}-2\arccos\left(\sqrt{\frac{l_{c}}{2r}}\right)$$

Similarly, for $\theta_{end}$ it holds

(5) $$l_{c}=2r\cos\left(\alpha \right)\cos\left(\theta_{end\;}-\alpha \right)$$

(6) $$\theta_{end\;}=\alpha +\arccos\left(\frac{l_{c}}{2r\cos\alpha }\right),\alpha \neq \pm 90{}^{\circ}$$

**Figure 5:**
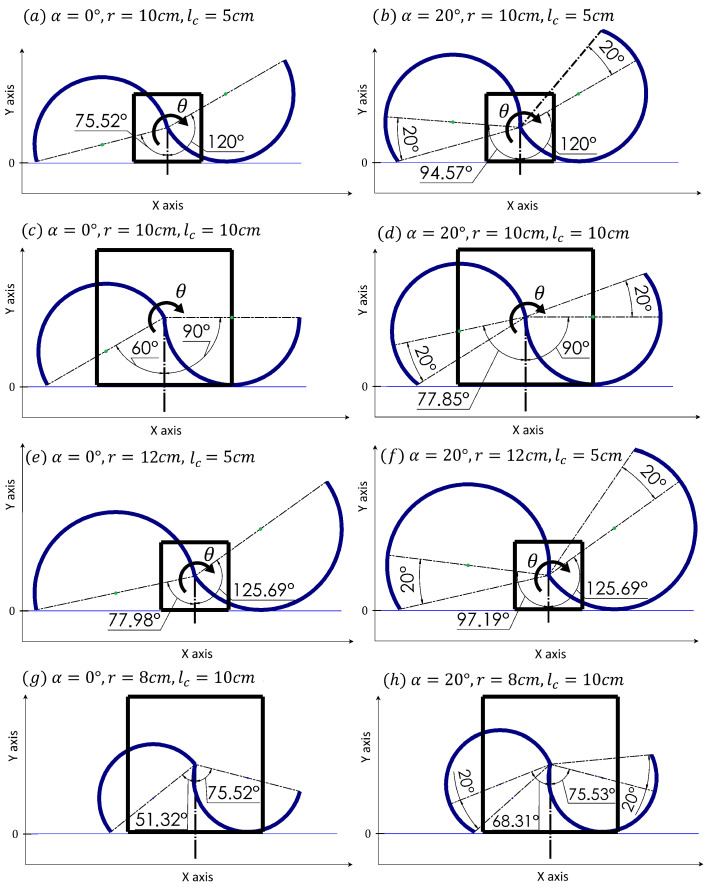
The influence of RHex leg parameters α, *r*, $l_{c}$ on $\theta_{start}$ and $\theta_{end}$.

The variation of $\theta_{start}$ and $\theta_{end}$ with varying $r,l_{c}$ and α are shown in Figure 5. It can be seen that changing any of these parameters has a significant impact on the leg surface that will participate in movement.

**Figure 6:**
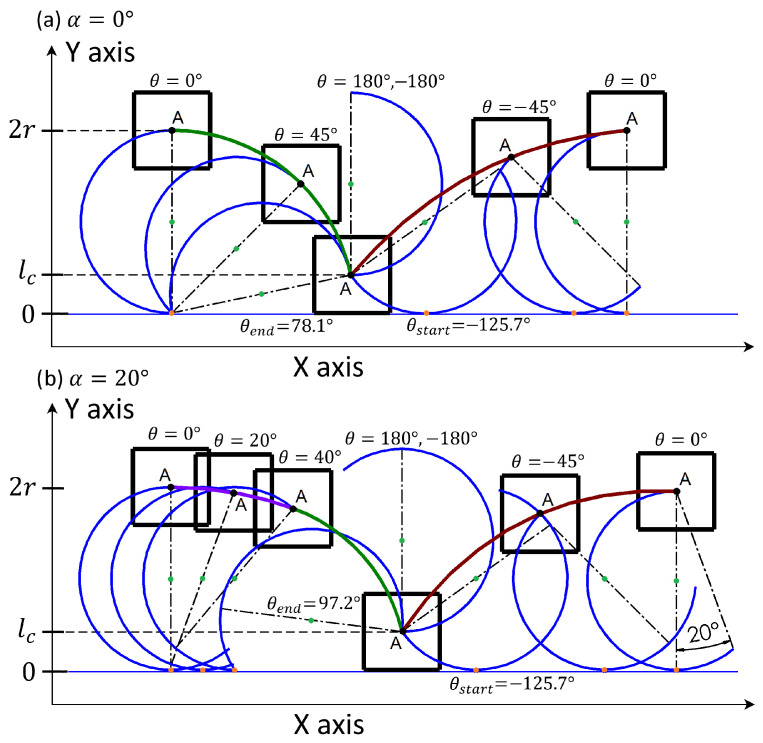
Framed motion of a single RHex robot leg for (**a**) α=0° and (**b**) α=20°.

We consider the crawling gait where the legs alter their positions in a cyclic manner over time, denoted by θ(t), by rotating at a constant velocity. This rotation propels the robot’s body along the X-axis (parallel to the ground in the direction the robot traverses) and the Y-axis (perpendicular to the ground, aligned with the direction of gravitational pull). The locomotion of the body with a single C-shaped leg through one complete revolution of the leg is depicted for α=0° and $\alpha \in \left[0,90{}^{\circ}\right]$ in Figure 6.

In the first case (see Figure 6a for α=0°), the system starts at the position where the contact point of the leg with the ground is located at the tip of the leg and the system pivots around the contact point until the chassis is in contact with the ground at leg position $\theta_{end}$. In that pivoting movement for $\theta \in [0{}^{\circ},\theta_{end\;}]$, the position x(θ) of the robot in the X-axis and y(θ) in the Y-axis are described by:

(7) $$x\left(\theta \right)=2r\sin\theta \left(t_{n}\right)$$

(8) $$y\left(\theta \right)=2r\cos\theta \left(t_{n}\right)$$

where time $t_{n}=nT,n\in N$, resulting from the sampling process with the period *T* and $t_{0}=0$, $\theta \in \left[0,\theta_{end}\right]$, $x\left(0{}^{\circ}\right)=0$, $y\left(0{}^{\circ}\right)=2r$. The position of the center of mass (Point A in Figure 3a) as a discrete signal can be described as

(9) $$\left[\begin{array}{c} x(t_{n})\\ y(t_{n}) \end{array}\right]=\left[\begin{array}{c} x\left(t_{n}-t_{n-1}\right)+2r\sin\theta \left(t_{n}\right)-2r\sin\theta \left(t_{n}-t_{n-1}\right)\\ 2r\cos\theta (t_{n}) \end{array}\right]$$

The pivoting motion ends when the chassis of the robot comes into contact with the ground and the robot’s leg detaches from the ground (as the leg rotates, see Point A in Figure 6a). The leg is then rotated over the robot body and leans on the ground in front of the chassis. It then starts another motion of the center of mass. When the robot leg is detached from the ground for $\theta \in \left[-180{}^{\circ},\theta_{start}\right]\cup \left[\theta_{end\;},180{}^{\circ}\right]$, the system becomes stationary in the so-called aerial phase. After making ground contact following the aerial phase, further rotation of the leg drives the system into a second ground phase with an ascending type of motion that corresponds to a cycloid (contact point of the leg with the ground is moving through the surface of the C-shaped leg towards its tip). Therefore, positions x(θ) and y(θ) can be described as

(10) $$x\left(\theta \right)=r\left(\theta \left(t_{n}\right)+180{}^{\circ}-\sin\left(\theta \left(t_{n}\right)+180{}^{\circ}\right)\right)=r\left(\theta \left(t_{n}\right)+180{}^{\circ}+\sin\theta (t_{n})\right)$$

(11) $$y\left(\theta \right)=r\left(1-\cos\left(\theta \left(t_{n}\right)+180{}^{\circ}\right)\right)=r\left(1+\cos\theta (t_{n})\right)$$

In that ascending movement for $\theta \in [\theta_{start\;},0{}^{\circ}]$, the position x(θ) of the robot in the X-axis and y(θ) in Y-axis are described as a discrete signal with a sampling period *T* using

(12) $$\left[\begin{array}{c} x(t_{n})\\ y(t_{n}) \end{array}\right]=\left[\begin{array}{c} x\left(t_{n}-t_{n-1}\right)+r\left(\theta \left(t_{n}\right)-\theta \left(t_{n}-t_{n-1}\right)+\sin\theta (t_{n})-\sin\theta (t_{n}-t_{n-1})\right)\\ r+r\cos\theta (t_{n}) \end{array}\right]$$

At the end of that phase (θ=0°), the whole process is repeated. Thus, one rotation of the leg in the single-leg system for α=0° can be divided into an aerial phase for $\theta \in \left[-180{}^{\circ},\theta_{start}\right]\cup \;\left[\theta_{end},180{}^{\circ}\right]$ and a ground phase for $\theta \in [\theta_{start},\theta_{end}]$. The ground phase can be further divided into a descending (pivoting) and ascending (cycloid) motion where the transition between them occurs at θ=0°.

For the second case with α>0° (see Figure 6b), the sequence is slightly changed. At the start it is not the tip of the leg that makes contact with the ground but it is the leg surface. The rotation of the leg causes a descending movement that corresponds to a cycloid. For θ=2α, the leg-ground contact point reaches the leg’s tip moving the system in a pivoting style of motion similar to when α=0° but the pivoting is with a smaller radius due to the enlarged part of the leg. After this point, the rest of the movement is identical to the first case. The descending cycloidal movement at the start and ascending cycloidal movement at the end phase of the cycle are fragments of the same cycloid.

By comparing both cases, one can observe that the basic types of the movements in both cases are the same and the only difference lies in the ranges of leg position at which specific types of motion occur. To be more specific, in both cases, the transition between the pivoting and cycloid type of motion in the ground phase occurs at θ=2α. By combining the obtained information, the X and Y axis crawling gait displacement of the center of mass of the RHex robot (after discretization with the sampling period *T*) can be described using

(13) $$\left[\;\begin{array}{c} x(t_{n})\\ y(t_{n}) \end{array}\;\right]=\left\{\begin{array}{c} \left[\;\begin{array}{c} x\left(t_{n}-t_{n-1}\right)+r\left(\theta \left(t_{n}\right)-\theta \left(t_{n}-t_{n-1}\right)+\sin\theta (t_{n})-\sin\theta (t_{n}-t_{n-1})\right)\\ r+r\cos\theta (t_{n}-t_{n-1}) \end{array}\;\right]\;\;\mathrm{if}\;\theta \left(t_{n}\right)\in \left[\theta_{start},2\alpha \right)\\ \left[\begin{array}{c} \;x\left(t_{n}-t_{n-1}\right)+2r\cos\alpha \left(\sin\left(\theta (t_{n})-\alpha \right)-\sin\left(\theta (t_{n}-t_{n-1})-\alpha \right)\right)\\ 2r\cos\alpha \cos\left(\theta (t_{n})-\alpha \right) \end{array}\;\right]\;\;\mathrm{if}\;\theta \left(t_{n}\right)\in \left[2\alpha ,\theta_{end}\right]\\ \left[\;\begin{array}{c} x(t_{n}-t_{n-1})\\ l_{c}\; \end{array}\right]\;\;\mathrm{if}\;\theta \left(t_{n}\right)\in \left(-180{}^{\circ},\theta_{start}\right)\cup \left(\theta_{end},180{}^{\circ}\right] \end{array}\right.$$

## 3. RHex Tripods Motion Profile for Walking/Running Scenario

For the RHex robot, various locomotion modes such as walking, running, turning, and climbing are achieved through the employment of predetermined periodic leg position setpoint functions. These functions are synchronized for each leg within one tripod (comprising legs 1-4-5 or 2-3-6, as illustrated in Figure 1A) and an alternated version for the opposite tripod. This coordination is widely recognized as the tripod gait, characterized by the robot maintaining a minimum of three points of contact with the ground at any given time. These contact points form a support triangle *S* (Figure 1B), which invariably encompasses the projection of the RHex robot’s center of mass (Figure 2), ensuring both dynamic and static stability. The rotation of both tripods is unidirectional, following a specific cyclic pattern. Within a single walking cycle, every leg of the RHex robot completes a full rotation, encompassing slow and fast swing phases. The slow swing phase facilitates the execution of a step, whereas the fast swing phase repositions the leg in preparation for the subsequent step. This alternating tripod stepping mechanism culminates in a stable walking pattern for the RHex robot. The alteration in position θ(t) of the RHex robot’s tripods (as depicted in Figure 1) during the slow swing phase induces a displacement akin to that described in Section 2. A notable distinction, however, lies in the variable rotation speed of the legs and the implementation of alternating motion profiles, which precludes any chassis–ground contact.

**Figure 7:**
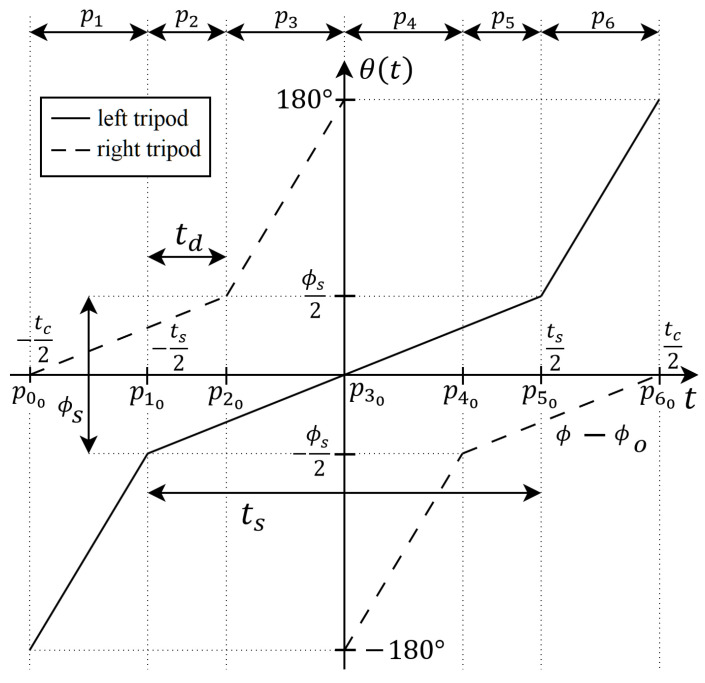
The motion profiles and essential parameters for the left and right tripods in a single walking cycle.

In the tripod gait, the legs of each corresponding tripod adjust their positions according to a cyclic time function θ(t), with a single cycle depicted in Figure 7. These leg position trajectories, often referred to as the ’Buehler clock’ or ’motion profiles of tripods’, define the robot’s kinematic behavior [33]. The trajectory θ(t) is characterized by several parameters: the period of the motion profiles $t_{c}$, the duty factor of each tripod within a cycle $t_{s}$, the angle covered during the slow swing phase $\phi_{s}$, with $\phi_{s}\in \left[0,180{}^{\circ}\right]$, and the motion profile offsets $\phi_{o}$. Typically, $t_{s}\in \left(0,t_{c}\right]$, but for faster movement, a duty factor in the range $t_{s}\in \left[\frac{t_{c}}{2},t_{c}\right]$ is advised. Each tripod undergoes slow and fast swing phases within a cycle, spanning angles $\phi_{s}$ and $360{}^{\circ}-\phi_{s}$, respectively, to complete a full rotation.

The optimal walking gait of the RHex robot can be attained through precise control of the parameters $t_{c}$, $t_{s}$, $\phi_{s}$, and $\phi_{o}$. Manipulating these values allows for the adjustment of the distance covered in a single walking cycle, modulation of the robot’s body turbulence along the Y-axis (as shown in Figure 1), and the timing of the double support phase ($t_{d}$ shown in Figure 7), where all six legs potentially make simultaneous ground contact during the slow swing phase. The extent of the double support phase $t_{d}$ is contingent upon the duty factors of the two tripods. A scenario with $t_{s}=\frac{t_{c}}{2}$ eliminates the double support phase entirely ($t_{d}=0$). The implementation of double support is particularly beneficial under conditions of heightened load on the leg drive motor or when enhanced stability is necessary, such as during transport of a payload by the robot. Nonetheless, prolonging the dual support phase inversely affects the robot’s locomotive speed, a detail further explored later in this study. The motion profile offset, denoted by $\phi_{o}$, adjusts the trajectory relative to the vertical (Figure 7) and is typically set to 0° in most applications.

Based on motion profiles presented in Figure 7 the rotation speed in fast swing (in the aerial phase) can be calculated as

(14) $${\dot{\theta }}_{F}\left(t\right)=\frac{{360{}^{\circ}-\phi }_{s}}{t_{c}-t_{s}}$$

And is the for the rotation speed in slow swing (ground phase):

(15) $$\dot{\theta_{S}}\left(t\right)=\frac{\phi_{s}}{t_{s}}$$

Thus, the rotation speed in time ${\dot{\theta }}_{L}\left(t\right)$ for the left and rotation speed in time ${\dot{\theta }}_{R}\left(t\right)$ for the right tripod in one cycle of the tripod gait can be presented, respectively, as:

(16) $${\dot{\theta }}_{R}\left(t\right)=\left\{\begin{array}{cc} {\dot{\theta }}_{S}\left(t\right), & t\in \left[p_{0_{0}},p_{2_{0}}\right]\cup \left[p_{4_{0}},p_{6_{0}}\right]\\ {\dot{\theta }}_{F}\left(t\right), & t\in (p_{2_{0}},p_{4_{0}}) \end{array}\right.$$

(17) $${\dot{\theta }}_{L}\left(t\right)=\left\{\begin{array}{cc} {\dot{\theta }}_{S}\left(t\right), & t\in (p_{1_{0}},p_{5_{0}})\\ {\dot{\theta }}_{F}\left(t\right), & t\in \left[p_{0_{0}},p_{1_{0}}\right]\cup \left[p_{5_{0}},p_{6_{0}}\right] \end{array}\right.$$

where ${p_{i}}_{0},\;i=1,\ldots ,6$ are time stamps of the $p_{i}$ phase end and $p_{i+1}$ phase start (see Figure 7).

In Table 1, a single Buehler clock cycle is segmented into six distinct phases, labeled as $p_{i},i=1,\ldots ,6$ in Figure 7. The duration of phases $p_{1},p_{3},p_{4},p_{6}$ is set at $\frac{t_{c}-t_{s}}{2}$, whereas phases $p_{2},p_{5}$ span a timeframe of $t_{s}-\frac{t_{c}}{2}$. This configuration establishes the temporal markers ${p_{i}}_{0},i=1,\ldots ,6$ that signify the conclusion of phase $p_{i}$ and the commencement of phase $p_{i+1}$ within a single walking cycle. Observations from a recorded RHex robot tripod gait, as documented in [34], reveal variations in the rotation speed of the tripods between successive movement phases. Specifically, the fast swing phases are attributed to the aerial phase, while the slow swing phases correlate with the ground contact phase of the tripod’s motion. Additionally, Table 1 delineates the type of movement along the Y-axis for the corresponding phases as witnessed in the recordings.

By analyzing Figure 7 and Table 1, the movement phases can be categorized into pairs $\left\{p_{1};p_{6}\right\}$, $\left\{p_{3};p_{4}\right\}$, and $\left\{p_{2};p_{5}\right\}$. Within these pairs, both the rotation speed of the specific tripods and the duration of the phases are identical. The pairs $\left\{p_{1};p_{6}\right\}$ and $\left\{p_{3};p_{4}\right\}$ facilitate the slow swing for the right and left tripods, respectively, with the primary distinction being the tripod that is active during each phase. Conversely, phases $p_{2}$ and $p_{5}$ correspond to the double support phases, during which both tripods engage in a slow swing and may simultaneously make contact with the ground.

Note that the left and right tripod in tripod gait rotate in the same direction (see Figure 7) to cause a forward displacement of the RHex robot. The direction of rotation of the tripods can be reversed to achieve a backward motion. However, it is not as optimal and stable as forward running and can be harmful to the leg drive motor because of sudden load increase when the leg starts to touch the ground.

## 4. RHex Incremental Kinematic Model for Walking in Flat Terrain

To obtain an incremental kinematic model of the RHex robot for walking gaits in flat terrain that uses alternating tripod motion profiles, some simplifying assumptions have been made:

- The leg has no mass—the RHex robot’s legs are a very small fraction of the total mass of the robot. Therefore, assuming a massless leg will not largely impact the motion mechanics of the system.
- No bending of the leg—in this analysis the C-shaped leg is considered to be a rigid body, despite the potential for leg deformation under load that can improve the robot’s mobility by functioning as a form of suspension. This aspect is particularly relevant for running gaits, where the RHex robot’s vertical (Y-axis) motion may exhibit distinctive characteristics. For instance, at high leg rotation speeds, the system may predominantly engage in ascending motion phases, where the combined effects of momentum and gravitational forces enable the robot to execute a series of jumps, minimizing the descending motion phases. However, in walking gaits, where the forces involved are considerably lower, the compliance of the legs does not significantly alter the system’s fundamental motion patterns. By modeling the legs as rigid bodies, the robot’s movement can be simplified to a combination of pivoting and cycloidal motions, which will be further explored as representing the foundational movement patterns of the RHex robot.
- No slipping between the robot’s legs and the ground.
- No change in the system mass.
- $\phi_{s}\in \left(0{}^{\circ},360{}^{\circ}\right],t_{s}\in \left[\frac{t_{c}}{2},t_{c}\right]$.

Within the framework of the tripod gait, the RHex robot can be conceptually simplified to a two-degree-of-freedom rigid body, outfitted with two semicircular legs that share a common axis of rotation. Each leg in this model epitomizes one half of the robot’s bipartite tripod mechanism. This reduction is justified by the dynamic stability inherent to the tripod gait, which, during locomotion across flat terrains, restricts the robot’s body displacement to the X and Y axes, as illustrated in Figure 1. The synchronized movement of the legs forming each tripod (effectively acting as a ’virtual leg’) consistently maintains three points of contact with the ground, effectively nullifying any rotational movement of the body. Consequently, it is reasonable to posit that the center of mass displacement in the actual RHex robot, when employing the tripod gait, mirrors that of its simplified counterpart.

**Figure 8:**
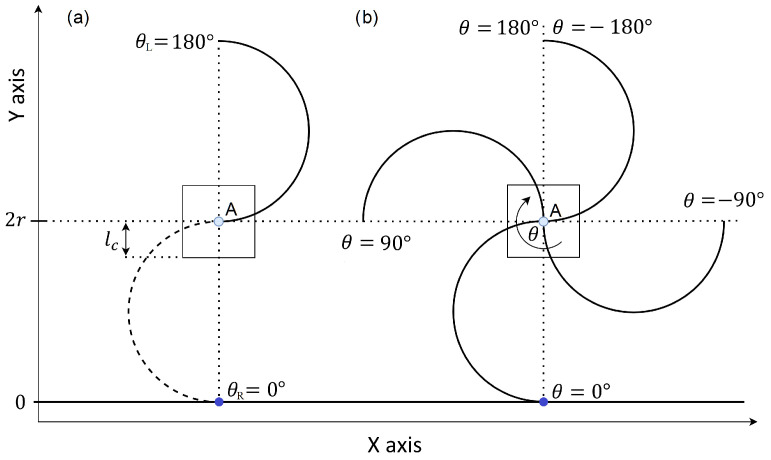
Kinematics of the simplified RHex robot with C-shaped leg for α=0° showing the (**a**) initial position of the legs in each cycle and (**b**) the position θ of the legs.

We develop a simplified model as depicted in Figure 8a, that serves as a partial representation of the RHex robot. This model comprises a square chassis, symbolizing the robot’s body, and a pair of C-shaped legs, each originating from a different tripod. The initial leg positions correspond to those outlined in the motion profiles (refer to Figure 7). The leg’s pivot point—where it attaches to the motor—is situated at Point A in Figure 8a. It’s crucial to note that for the model to be applicable, all legs of the RHex robot must share a common Y-axis level at their pivoting points. The blue dot in the figure denotes the contact point where the leg meets the ground, represented by a solid black line at zero meters elevation. The X-axis, running horizontally and parallel to the ground, signifies the direction of the robot’s forward and backward traversal, while the Y-axis, perpendicular to the ground, aligns with the gravitational pull. These axes align with those presented in Figure 1.

As the leg rotates clockwise (as shown in Figure 8b), the ground contact point shifts, prompting the system to displace. The construction attributes of the RHex robot, such as the leg radius *r*, the distance $l_{c}$ from the leg’s pivot Point A to the chassis bottom, and the leg extension α, are described using the same parameters introduced in Section 2. The range $\theta \left(t_{n}\right)\in \left[\theta_{start},\theta_{end}\right]$ within which the robot’s leg maintains ground contact is defined in a manner analogous to that in Section 2.

Initial positions of the right and left leg (each represent corresponding tripod of the RHex robot) in the simplified system can be described using:

(18) $$\theta_{R}\left(t_{0}\right)=0$$

(19) $$\theta_{L}\left(t_{0}\right)=-180{}^{\circ}$$

Using the initial position of the legs, the initial position of the robot’s center of mass is determined as:

(20) $$x\left(t_{0}\right)=x_{0}=0$$

(21) $$y\left(t_{0}\right)=y_{0}=2r$$

**Figure 9:**
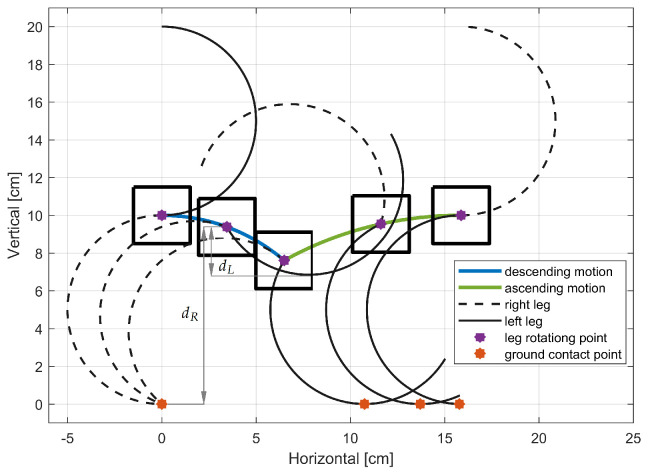
Tripod gait half cycle visualization for r=5 cm, α=0°, $\phi_{s}=90{}^{\circ}$ and $t_{s}=\frac{t_{c}}{2}$.

To prevent the robot’s chassis from contacting the ground during the tripod gait, alternating motion profiles are employed. The trajectories for $t\in (0,\frac{t_{c}}{2})$ and $t\in (\frac{t_{c}}{2},t_{c})$, as illustrated in Figure 7, mirror each other irrespective of the specific tripod in action, rendering them as odd functions. Consequently, the robot’s movement is characterized by a series of half-cycle displacements. Furthermore, as elucidated in Section 2, the locomotion of the RHex robot when operating with a single leg encompasses both pivoting and cycloidal motions, as delineated by Equations (9) and (12), respectively, [1]. Building on these observations, it can be inferred that the movement of a bipedal configuration in the RHex robot consists of a cyclic pattern of ascending and descending motions. To gain a clearer understanding of the RHex robot’s displacement during a half-cycle of the tripod gait, a detailed visualization is provided in Figure 9. For the initial leg positions depicted in Figure 8a, the legs adjust their positions following the trajectory outlined in Figure 7, with parameters set to r=5 cm, α=0°, $\phi_{s}=90{}^{\circ}$ and $t_{s}=\frac{t_{c}}{2}$.

Initially, the system pivots on the right leg while the left leg rotates freely in the air. At a certain instance, both legs momentarily make contact with the ground, as depicted in the visualization at the lowest central position. Subsequently, the right leg loses ground contact, and the system’s progression is driven by the left leg’s motion, albeit in a cycloidal fashion. By the conclusion of the half-cycle’s visualization, the system reverts to a state akin to the initial condition, albeit with the left and right legs’ positions interchanged. This sequence recurs twice within a single tripod gait cycle, culminating with the legs reverting to their original positions ($\theta_{L}\left(t=t_{c}\right)=180{}^{\circ}$ and $\theta_{R}\left(t=t_{c}\right)=360{}^{\circ}$). The determining factor of which tripod is engaged with the ground, thereby facilitating displacement, hinges on the greater value of d(t)—the distance from the leg’s pivot point (illustrated by the purple dot in Figure 9) to the leg’s furthest extremity toward the ground along the Y-axis. For each tripod, d(t) is contingent upon the current position of its ’virtual leg’ θ(t), and thus, is time-dependent. A leg is considered in contact with the ground when its d(t) is equal to or surpasses that of the alternate virtual leg. The distance $d(t_{n})$ resulting from the sampling process with the period *T* for each virtual leg is defined as follows:

(22) $$d\left(t_{n}\right)=\left\{\begin{array}{cc} r-r\cos\theta (t_{n}) & \;\mathrm{if}\;\theta \left(t_{n}\right)\in \left[\theta_{start\;},2\alpha \right]\\ 2r\cos\left(\alpha \right)\arccos\left(\theta (t_{n})-\alpha \right) & \;\mathrm{if}\;\theta \left(t_{n}\right)\in \left(2\alpha ,\theta_{end\;}\right]\\ l_{c} & \;\mathrm{if}\;\theta \left(t_{n}\right)\in \left(-180{}^{\circ},\theta_{start\;}\right)\cup \left(\theta_{end\;},180{}^{\circ}\right] \end{array}\right.$$

Designation of the tripod responsible for the movement and its current position at any given time is derived from comparing the distance $d(t_{n})$ of both legs as

(23) $$\theta_{G}\left(t_{n}\right)=\left\{\begin{array}{cc} \theta_{L}\left(t_{n}\right), & \;\mathrm{if}\;d_{R}\left(t_{n}\right)⩽d_{L}\left(t_{n}\right)\\ \theta_{R}\left(t_{n}\right), & \;\mathrm{if}\;d_{R}\left(t_{n}\right)>d_{L}\left(t_{n}\right) \end{array}\right.$$

where $\theta_{L}\left(t_{n}\right)$ is the current position of the legs in the left tripod with distance $d_{L}\left(t_{n}\right)$, $\theta_{R}\left(t_{n}\right)$ is the current position of the legs in the right tripod with distance $d_{R}\left(t_{n}\right)$, see Figure 9. By combining Equation (13) of one-legged RHex robot system with Equation (23), the X and Y displacement of the center of mass of the RHex robot (after discretization with sampling period *T*) for tripod gait can be described as

(24) $$\left[\begin{array}{c} x(t_{n})\\ y(t_{n}) \end{array}\right]=\left\{\begin{array}{c} \left[\begin{array}{c} x\left(t_{n}-t_{n-1}\right)+r\left(\theta_{G}\left(t_{n}\right)-\theta_{G}\left(t_{n}-t_{n-1}\right)+\sin\theta_{G}\left(t_{n}\right)-\sin\theta_{G}\left(t_{n}-t_{n-1}\right)\right)\\ r+r\cos\theta_{G}\left(t_{n}\right) \end{array}\right]\;\mathrm{if}\;\theta_{G}\left(t_{n}\right)\in \left[\theta_{start\;},2\alpha \right)\\ \left[\begin{array}{c} x\left(t_{n}-t_{n-1}\right)+2r\cos\alpha \left(\sin\left(\theta_{G}\left(t_{n}\right)-\alpha \right)-\sin\left(\theta_{G}\left(t_{n}-t_{n-1}\right)-\alpha \right)\right)\\ 2r\cos\alpha \cos\left(\theta_{G}\left(t_{n}\right)-\alpha \right) \end{array}\right]\;\;\mathrm{if}\;\theta_{G}\left(t_{n}\right)\in \left[2\alpha ,\theta_{end\;}\right]\\ \left[\begin{array}{c} x(t_{n}-t_{n-1})\\ l_{c} \end{array}\right]\;\;\mathrm{if}\;\theta_{G}\left(t_{n}\right)\in \left(-180{}^{\circ},\theta_{start\;}\right)\cup \left(\theta_{end\;},180{}^{\circ}\right] \end{array}\right.$$

**Figure 10:**
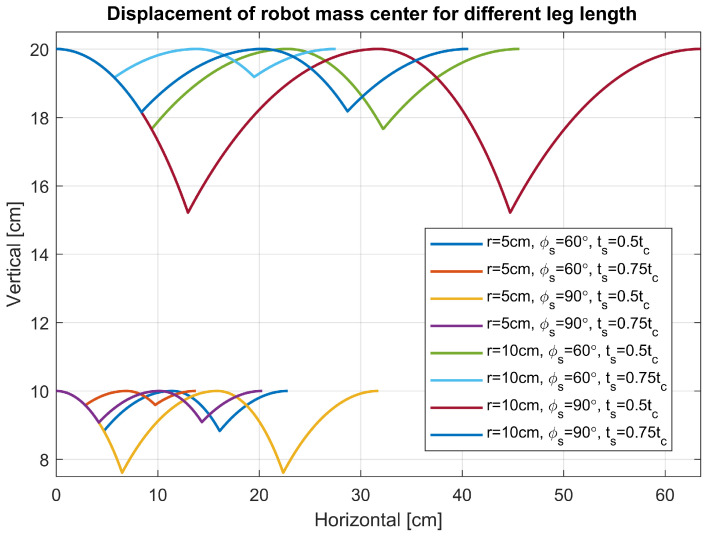
Comparison of robot displacement in X and Y axis for different *r*, $t_{s}$ and $\phi_{s}$.

The developed model featuring α=0°, variable $t_{s}$, *r*, and $\phi_{s}$ was simulated to evaluate the impact of these parameters on the robot’s locomotion. The results are presented in Figure 10. Consistent with the model’s premises, the robot’s displacement embodies the movement types previously delineated. Notably, the displacement along the X-axis and Y-axis is directly influenced by the leg’s radius, highlighting that even minor adjustments to the leg’s radius can significantly affect the robot’s operational range—a crucial factor in defining its potential applications.

To alter the robot’s walking, adjustments to other parameters are necessary. For instance, augmenting $t_{s}$ within the motion profile diminishes the X-axis displacement while concurrently reducing the oscillation amplitude of the robot’s virtual center of mass along the Y-axis. This reduction is particularly advantageous when employing optical sensors. Similar effects are observed with a decrease in $\phi_{s}$. Therefore, by simultaneously increasing $t_{s}$ and decreasing $\phi_{s}$, comparable walking can be achieved through diverse motion profiles. This interdependency offers valuable insights for designing varied gaits tailored to specific tasks such as running or load-bearing, where the duration of double support phases may necessitate adjustment.

## 5. Experimental Validation of the RHex Walking Model

To corroborate the kinematic model presented in the preceding section, the creation of an experimental test bed congruent with the model’s premises was imperative. A critical aspect of this setup was the constriction of the system’s degrees of freedom exclusively in the X and Y axes. This limitation was essential to ensure no extraneous resistance was introduced, thereby allowing for an accurate emulation of the motion of the mobile robot’s center of mass.

**Figure 11:**
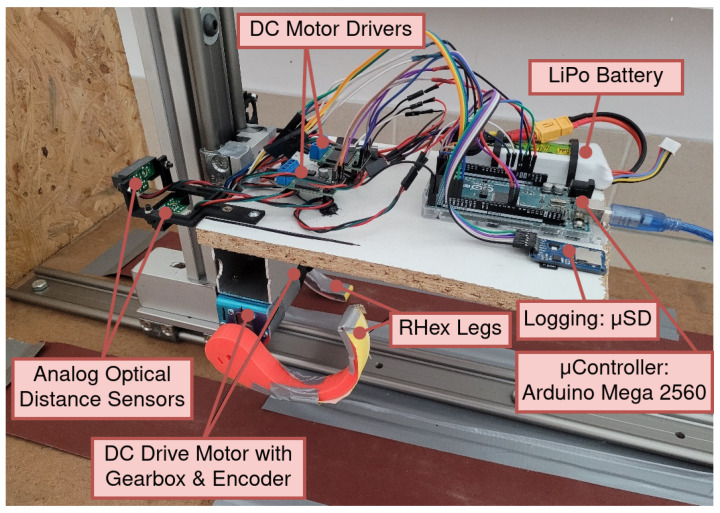
Experimental test bed fabricated to emulate and validate the kinematic model.

For the validation of the kinematic model, the experimental test bed depicted in Figure 11 was meticulously designed and fabricated. The test bed incorporates a dual set of linear guideway blocks and rails, commonly found in CNC machinery, configured to permit motion along the X- and Y-axes while constraining movement and rotation across other axes. To ensure minimal resistance and friction, lubricants and bearings were integrated within the blocks. A subsystem comprising a pair of RHex robot legs, representing the mobile robot, was mounted onto this bespoke structure. To faithfully replicate the robot’s control mechanisms, components identical to those utilized in the FLHex robot, as documented in [27], were employed. This setup includes an Arduino Mega 2560 (Arduino.cc Corp.) microcontroller and a Pololu VNH5019 (Pololu Corp., Las Vegas, NV, USA) motor driver for control, a high-torque 12V DC motor Pololu 37Dx70L (Pololu Corp., Las Vegas, NV, USA) series with a 50:1 gearbox for actuation, and a quadrature magnetic encoder for position sensing. The leg position control system of the RHex robot is governed by a fractional-order PID (FOPID) controller with optimally derived coefficients [36] and aims to maintain the leg positions in tight alignment with the predefined motion profiles through a series of carefully executed steps.

The leg center positions during rotation were estimated using analog optical distance sensors: a Sharp GP2Y0A41SK0F (Sharp Corp., Osaka, Japan) for the Y-axis and a Sharp GP2Y0A21YK0F (Sharp Corp., Osaka, Japan) for the X-axis. To mitigate sensor noise, a quadratic regression was applied over a 100-sample window. Both sensors were interfaced with an Arduino Mega, which was collecting and filtering this data. Arduino board was receiving FOPID controller outputs from a laptop running MATLAB 2022b where horizontal and vertical legs positions were calculated and visualized. For these experiments, the leg radius was set at 5 cm, designed to meet the model’s stipulated requirements. As shown in Figure 11, the motor shaft and leg end form a semicircle. A sandpaper was used as a walking surface to prevent slippage. Additionally, the leg was engineered to minimize bending.

**Figure 12:**
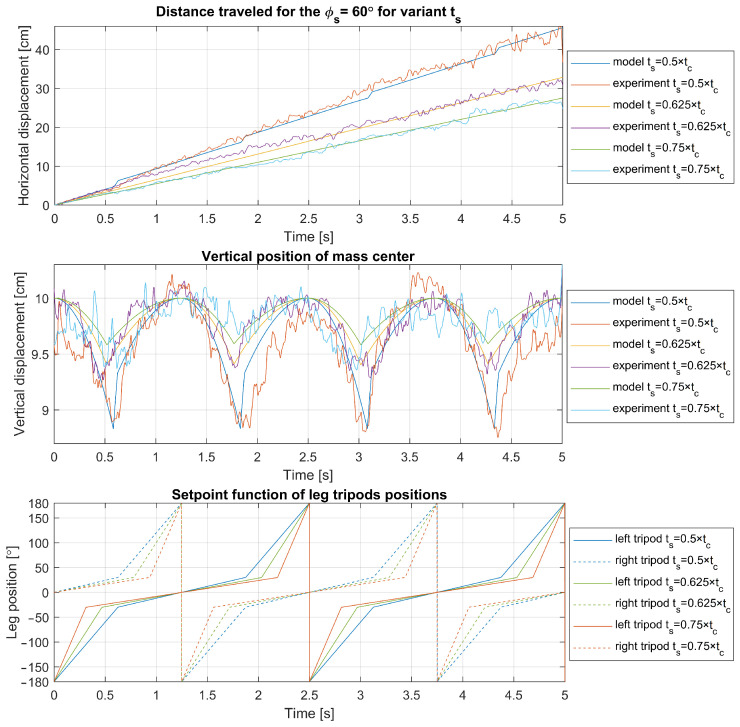
Experimental validation of model for $t_{c}=2.5$ s, $\phi_{o}=0{}^{\circ}$, $\phi_{s}=60{}^{\circ}$ with varying $t_{s}$.

**Figure 13:**
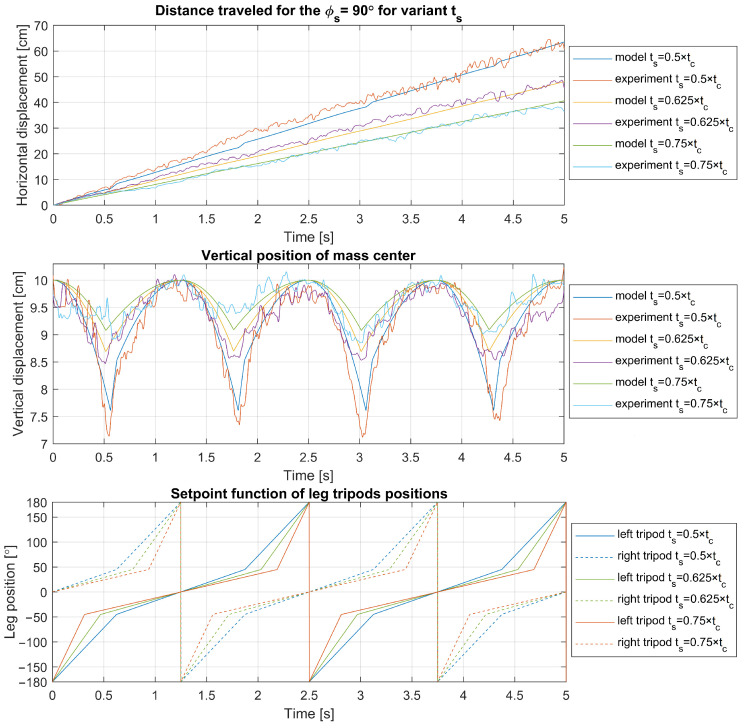
Experimental validation of model for $t_{c}=2.5$ s, $\phi_{o}=0{}^{\circ}$, $\phi_{s}=90{}^{\circ}$ with varying $t_{s}$.

Experimental results are juxtaposed with model predictions for legs with parameters r=5 cm, $\phi_{s}=60{}^{\circ}$ in Figure 12, and with parameter $\phi_{s}=90{}^{\circ}$ in Figure 13. The results are with varying $t_{s}$. Each figure delineates the horizontal and vertical displacements alongside the corresponding leg positions over time. Notably, the most significant discrepancies were observed in height changes, although these deviations were minimal relative to the leg’s size. These differences could partially result from friction between the guideway block and rail or errors in optical distance measurement. Some degree of unavoidable leg bending may also contribute to these discrepancies. Crucially, the experimental travel distances align with the model’s predictions, affirming the kinematic model’s applicability to the walking/running control logic of the robot and its overall accuracy.

## 6. Results and Discussion

A significant observation in both the experimental tests and kinematic model prediction for $t_{s}=0.5t_{c}$ is the abrupt increase in both horizontal and vertical displacements observed immediately following the transition between tripod sets during ground contact (shifting from the declining phase of one tripod’s step to the ascending phase of the other). This transition results in an augmented displacement for the robot over a larger number of steps to some degree.

**Figure 14:**
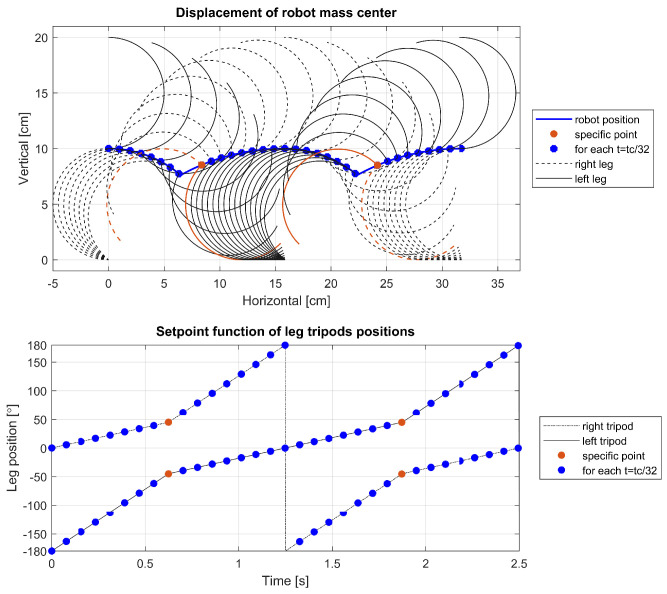
Displacement of the robot for $t_{s}=0.5t_{c}$.

**Figure 15:**
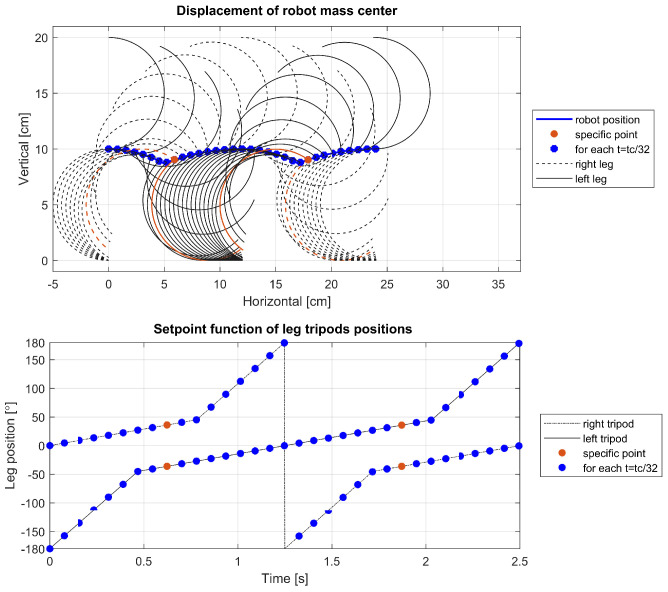
Displacement of the robot for $t_{s}=0.625t_{c}$.

**Figure 16:**
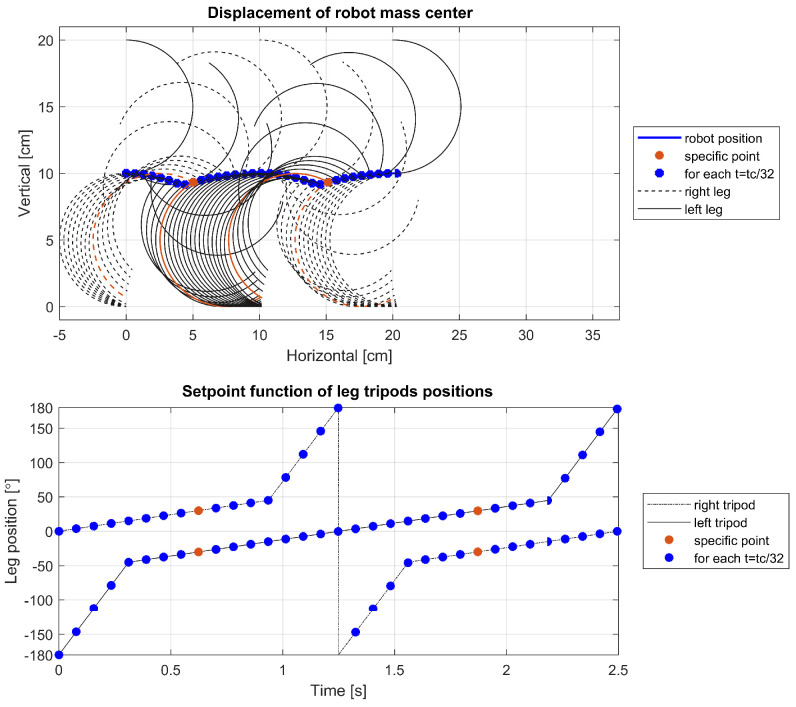
Displacement of the robot for $t_{s}=0.75t_{c}$.

To delve deeper into the cause of this phenomenon, further visualizations were conducted for a leg with parameters r=5 cm, α=0°, $t_{c}=2.5$ s, and varying $t_{s}$. The visualizations are showcased in Figure 14, Figure 15 and Figure 16 and depict the robot’s displacement during a single step cycle in the walking gait for $t_{s}=0.5t_{c}$, $t_{s}=0.625t_{c}$, and $t_{s}=0.75t_{c}$, respectively, with the leg positions visualized at equal time intervals of $t=0.03125t_{c}$. Notably, Figure 14 illustrates that around the 1.8-second, the points are significantly more spaced out compared to other instances, indicating a higher velocity during these periods as evidenced in Figure 12 and Figure 13. Upon examination of the visualized leg positions, it becomes apparent that in this scenario, the tripod designated for the aerial fast swing phase inadvertently makes ground contact, while the other tripod, which is supposed to propel the system through its motion and maintain ground contact, is detached. Such an occurrence is undesirable, as it prevents the leg from executing its intended function during that movement phase. Furthermore, an increased rotation speed of the leg upon ground contact may pose a risk of damage or accelerated wear to the robot’s drive components.

**Figure 17:**
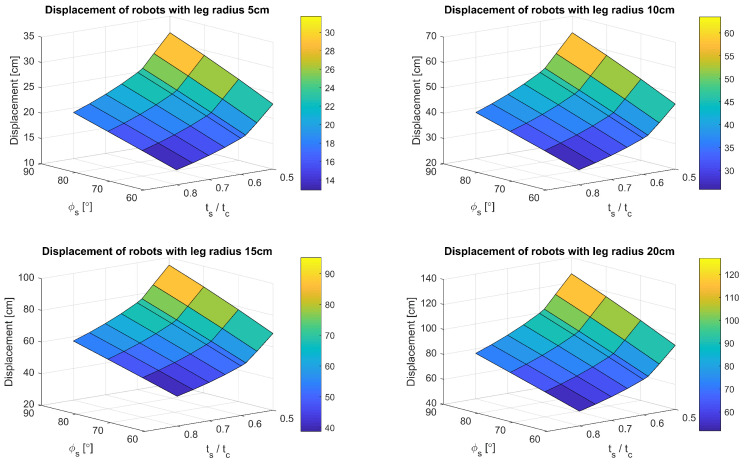
Comparison of the influence of $t_{s}$ and $\phi_{s}$ on the distance traveled by the robot for different leg radius.

To ascertain which aspects of the robot’s design or motion profiles might lead to the aforementioned undesirable occurrences, additional simulations were conducted. The initial focus was on specific parameters, setting α=0° while varying *r*, $t_{s}$, and $\phi_{s}$. The robot’s displacement along the X-axis during a single walking gait cycle served as the criterion for identifying instances of the undesired event, as such occurrences would typically manifest as a noticeable increase in displacement. The findings are documented in Figure 17. In all scenarios with α=0°, variations in *r* or $\phi_{s}$ resulted in a linear alteration of the X-axis displacement per cycle. Conversely, a non-linear response was observed for $t_{s}$, particularly when $t_{s}<0.585t_{c}$, where the X-axis displacement significantly increased, confirming the presence of the undesired event upon reviewing the leg positions in these instances.

**Figure 18:**
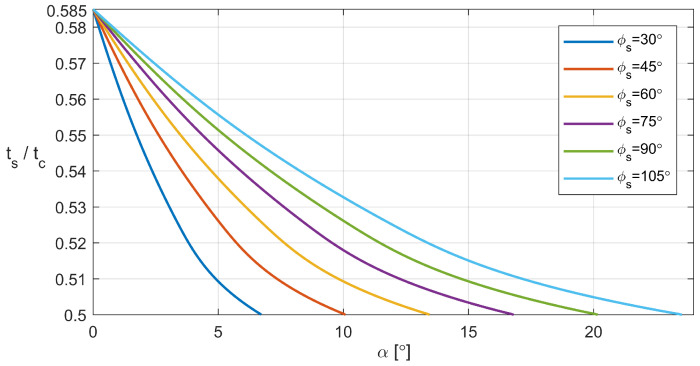
Minimal value of α for specified dependency of $\frac{t_{s}}{t_{c}}$ where legs in aerial phase do not make contact with the ground.

However, the situation grows more intricate with α>0°, revealing that the likelihood of the undesired event is influenced by a combination of α, $t_{s}$, and $\phi_{s}$. To circumvent this event, reference to a supplementary graph, illustrated in Figure 18, is recommended. Utilizing this graph involves selecting α and $t_{s}$ values such that their corresponding point on the graph resides on or above the line designated for the chosen $\phi_{s}$ within the motion profile.

## 7. Conclusions

In this study, we have comprehensively delineated the gait of RHex-type robots, highlighting the pivotal parameters influencing their locomotion. The investigative efforts culminated in the formulation of a kinematic iterative model, tailored for the gait control of such hexapod robots. This model’s fidelity was substantiated through rigorous experimental validations conducted on a specially designed test bench. Comparative analyses between the model and experimental outcomes revealed the manifestation of specific undesired phenomena under certain conditions dictated by the robot’s leg design and motion profile parameters. Crucially, these insights facilitated the creation of a heuristic graph, poised to guide the optimization of the RHex robot’s running gaits in forthcoming control strategies. By judiciously adjusting the gait parameters, it becomes feasible to tailor the kinematics to suit varying double support duration, catering to the robot’s immediate operational requirements. Nonetheless, it is imperative to acknowledge a fundamental compromise: enhancing the robot’s velocity, particularly in running gaits, invariably introduces increased oscillations along the Y-axis. This phenomenon could potentially compromise the accuracy of concurrent measurements, underscoring a critical consideration in the pursuit of elevated movement speeds. The forthcoming phase of this research will be dedicated to a comprehensive analysis of the RHex robot’s locomotion across terrains of heterogeneous characteristics, encompassing surfaces such as sand and the transitional zones from shorelines to aquatic environments. A focal point of the investigation will be the exploration of how the robot’s leg material properties influence the incidence of slippage between the leg and the terrain, thereby affecting the robot’s dynamic performance. Particular attention will be given to the impact of the flexibility and texture of the robot’s C-shaped legs, which play a pivotal role in its movement, on its interaction with diverse ground conditions. This in-depth examination aims to elucidate the intricate relationship between the robot’s structural design and its adaptability to complex environmental challenges. The authors have also initiated the integration of a neuromorphic walking controller into the robotic framework described within the article.

## Figures and Tables

**Table 1:** Motion phases $p_{i},i=1,2,\ldots ,6$ of the RHex robot in tripod alternating gait for walking cycle $\left(n_{j},j=0,1,\ldots \right)$.

Phase $p_{i}$ of Motion	Left Tripod Motion Type	Right Tripod Motion Type	Tripod Responsible for Movement	Robot Movement in Y Axis	Time Stamp of Phase End ${p_{i}}_{0}$ for Each Walking Cycle
$p_{1}$	Fast swing	Slow swing	Right	Descending	$n_{j}t_{c}+\left(t_{c}-t_{s}\right)/2$
$p_{2}$	Slow swing	Slow swing	Transition from right to left	From descending to ascending	$n_{j}t_{c}+t_{s}/2$
$p_{3}$	Slow swing	Fast swing	Left	Ascending	$n_{j}t_{c}+t_{c}/2$
$p_{4}$	Slow swing	Fast swing	Left	Descending	${(n_{j}+1)t}_{c}-t_{s}/2$
$p_{5}$	Slow swing	Slow swing	Transition from left to right	From descending to ascending	$n_{j}t_{c}+\left(t_{c}+t_{s}\right)/2$
$p_{6}$	Fast swing	Slow swing	Right	Ascending	$\left(n_{j}+1\right)t_{c}$

## Data Availability

Data are contained within the article.
